# Quantification of paravalvular leaks associated with TAVI implants using 4D MRI in an aortic root phantom made possible by the use of 3D printing

**DOI:** 10.3389/fcvm.2023.1083300

**Published:** 2023-01-19

**Authors:** Philipp Aigner, Eleonora Sella Bart, Sebastiano Panfili, Tito Körner, Markus Mach, Martin Andreas, Markus Königshofer, Simone Saitta, Alberto Redaelli, Albrecht Schmid, Francesco Moscato

**Affiliations:** ^1^Center for Medical Physics and Biomedical Engineering, Medical University of Vienna, Vienna, Austria; ^2^Ludwig Boltzmann Institute for Cardiovascular Research, Vienna, Austria; ^3^Department of Electronics, Information, and Bioengineering, Politecnico di Milano, Milan, Italy; ^4^Department of Cardiac Surgery, Medical University of Vienna, Vienna, Austria; ^5^Austrian Cluster for Tissue Regeneration, Vienna, Austria

**Keywords:** 3D printing, TAVI, paravalvular leaks, 4D MRI, ultrasound

## Abstract

**Introduction:**

Transcatheter aortic valve implantation (TAVI) has become an alternative to surgical replacement of the aortic valve elderly patients. However, TAVI patients may suffer from paravalvular leaks (PVL). Detecting and grading is usually done by echocardiography, but is limited by resolution, 2D visualization and operator dependency. 4D flow magnetic resonance imaging (MRI) is a promising alternative, which did not reach clinical application in TAVI patients. The aim of this study was applying 3D printing technologies in order to evaluate flow patterns and hemodynamics of PVLs following TAVI, exploiting 4D flow MRI and standard ultrasound.

**Materials and methods:**

An MR-compatible, anatomically left ventricle, aortic root, and ascending aorta model was fabricated by combining 3D-printed parts and various soft silicone materials to match physiological characteristics. An Abbott Portico™ valve was used in continuous antegrade flow (12–22 l/min), retrograde flow with varying transvalvular pressures (60–110 mmHg), and physiological pulsatile hemodynamics (aortic pressure: 120/80 mmHg, cardiac output: 5 l/min) Time-resolved MR measurements were performed above and below the TAVI stent and compared with color Doppler ultrasound measurements in exactly the same setup.

**Results:**

The continuous antegrade flow measurements from MRI largely agreed with the flowmeter measurements, and a maximum error of only 7% was observed. In the retrograde configuration, visualization of the paravalvular leaks was possible from the MR measurements, but flow was overestimated by up to 33%. The 4D MRI measurement in the pulsatile setup revealed a single main PVL, which was also confirmed by the color Doppler measurements, and velocities were similar (2.0 m/s vs. 1.7 m/s).

**Discussion:**

4D MRI techniques were used to qualitatively assess flow in a patient-specific, MR-compatible and flexible model, which only became possible through the use of 3D printing techniques. Flow patterns in the ascending aorta, identification and quantification of PVLs was possible and the location and extent of PVLs were confirmed by ultrasound measurements. The 4D MRI flow technique allowed evaluation of flow patterns in the ascending aorta and the left ventricle below the TAVI stent with good results in identifying PVLs, demonstrating its capabilities over ultrasound by providing the ability to visualize the paravalvular jets in three dimensions at however, additional expenditure of time and money.

## 1. Introduction

In the last decade, transcatheter aortic valve implantation (TAVI) has become an alternative procedure to surgical replacement of the aortic valve for the treatment of aortic valve stenosis (AS), that affects between 2 and 4% of the world population over 75 years of age (1). Paravalvular leakage (PVL) is one of the most critical problems related to TAVI (2). Moderate and severe PVLs have already been associated with an increased short and long-term mortality (3, 4) and even mild paravalvular regurgitations have been linked to poor outcomes (5–7). Therefore, the long-term effects of PVL after TAVI still need to be analyzed, as they could be crucial for the extension of TAVI therapy to younger patients and of great prognostic importance.

Flow patterns in the cardiovascular system play an important role not only for cardiac efficiency issues (8), but also in the development of complications and adverse events in different devices like left atrial appendage occlusion (9), ventricular assist devices (10) or malfunctions like aortic insufficiencies (11). TAVI valves are certainly no exception, though there are still some hurdles to jump and better understand the interaction here.

The detection, grading and tracking of PVL is mostly based on color Doppler echography, which has several limitations: it is operator dependent, allows only 2D visualization and the quality of the images is limited and low due to the technical limitation of the travel time of the ultrasound waves. 4D magnetic resonance imaging (MRI) is a promising alternative that is already being used for *in vivo* and *in vitro* evaluations of heart valves, but has not yet been studied in depth for applications related to TAVI and PVL.

The aim of this study was to evaluate the hemodynamics, to visualize forward and backflow and to quantify PVLs of a TAVI in a single test circuit using 4D flow MRI, ultrasound imaging and flowmeter measurements.

## 2. Materials and methods

### 2.1. Aortic arch and left ventricular phantom

**FIGURE 1:**
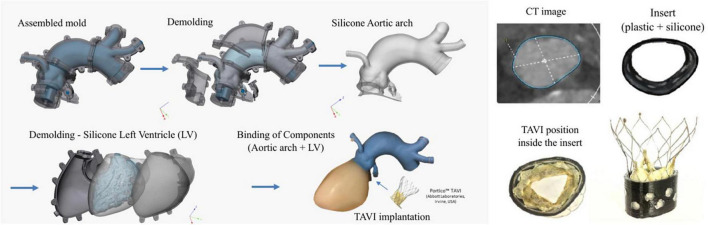
Three-dimensional representation of the phantom and molding process steps **(left)** and details of the CT image of the aortic annulus and fabrication of two layer insert with the transcatheter aortic valve implantation (TAVI) positioned within the insert **(right)**.

A cardiovascular model was fabricated using silicone casting (see Figure 1, left panel): a left ventricle and an aorta including the sinuses of Valsalva, coronary artery branches, brachiocephalic artery, and left common carotid artery. Details on segmentation, mold creation and 3D printing can be found elsewhere (12). The patient specific geometry was attained from the CT scan of a 71-year-old patient with severe aortic regurgitation. The patient was chosen given the elliptical shape of his aortic root, which, according to literature, is the main reason for PVLs together with aortic root/leaflet calcifications. Molds were designed for the left ventricle and the aorta consisting of multiple outer parts and removable inner cores, all printed in resin (Clear Resin V4, Formlabs Inc., Somerville, MA, USA) *via* a stereolithographic 3D printer (Formlabs Form 2, Formlabs Inc., Somerville, MA, USA). For the left ventricle, a model produced from translucent two-component silicone (Kiwosil RTV-2 S35, Farben Wolf GmbH, Vienna, Austria) with a Shore A hardness value of 35 was used. The ascending aorta model was fabricated from a two-component silicone (Smooth-Sil™ 950 with the addition of 5% Silicone Thinner™, Smooth-On Inc, Macungie, PA, USA) with a Shore A hardness of 50 and a tensile strength of 5 N/mm^2^ to match the arterial compliance. Finally, the compliance of the model was determined by applying defined volumes and accompanying pressure measurements.

The silicone ventricle model did not include the valve leaflets, and to correctly represent the annulus geometry, a ring insert was designed to match the actual annulus shape found in CT images (see Figure 1, right panel). The 17 mm long insert consisted of an 0.8 mm thick outer part produced from polylactic acid (PLA) by filament 3D printing (Anycubic i3 Mega, Anycubic, Frankfurt, Germany). The ring was internally coated with a 1 mm layer of silicone (Kiwosil RTV-2 S35, Farben Wolf GmbH, Vienna, Austria), in order to allow the TAVI stent to adhere to the silicone covered wall. Good mechanical connection between the two layers was achieved by means of circle-shaped recess to prevent slippage. Finally, this insert effectively prevented the model from deforming and becoming round during the diastolic phase or retrograde pressurization.

A self-expanding transcatheter 27 mm artificial aortic valve (Portico™, Abbott Laboratories, Irvine, USA) was implanted using the proper delivery system (Abbott Laboratories, Irvine, CA, USA) (13). Both the TAVI stent and the phantom were marked with reference points to ensure reproducibility of the position and orientation of the valve within the phantom. To avoid dislocation of the prosthetic valve during testing, the TAVI stent was additionally secured to the aortic root with six sutures, as the soft tissue properties of valve tissue was missing in the model.

### 2.2. Continuous antegrade and retrograde flow setup

Two circuits were developed for MRI examination, which allowed continuous flow (antegrade and retrograde) and pulsatile flow. The circuits were filled with a commonly used blood analog fluid consisting of water and glycerol (60% water and 40% glycerol) with density and viscosity (3.72 cP) values very similar to those of blood (14).

**FIGURE 2:**
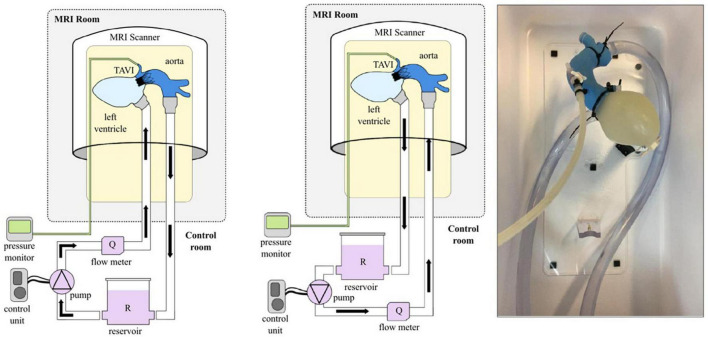
Schematic representation of the setup in the case of antegrade **(left)** and retrograde **(middle)** flow and photograph of the mock loop **(right)**.

The setup for continuous antegrade and retrograde perfusion was essentially identical. A pump in the control room was used to pump the blood analog into the MRI room through the cardiac model and back *via* a total of 16 m of 19 mm polyvinyl chloride (PVC) tubing (see Figure 2) at peak systolic flow rates. To monitor the pressure in the model, a pressure line was placed from the coronary artery into the control room (TruWave disposable pressure transducers, Edwards Lifesciences Corporation, Irvine, California and Viridia CMS Anesthesia Monitor with Model 66S M1166A Module Rack, Hewlett Packard, Palo Alto, CA, USA). Outside the MR room, flows were measured by means of a transit time flowmeter (Flowprobe H16XL, HT110R Transonic systems, New York, NY, USA) and the backflow was collected in a reservoir.

Since different hemodynamic working points were chosen for the antegrade and retrograde flows (see Table 1), different pumps had to be chosen here as well. A bilge pump (Jabsco Commercial Duty Water Puppy, Xylem Inc., New York, USA) was used for the large continuous forward flow of up to 22 l/min. For the retrograde flow a centrifugal pump (BioPump BPX-80, Medtronic Biomedicus Inc., Minnesota, USA) was used to apply physiological transvalvular pressures of 60–90 mmHg.

**TABLE 1:** MR encoding rates and acquisition times used in continuous flow acquisitions (longitudinal means the direction parallel to the direction of flow in the ascending aorta).

Antegrade continuous flow				Retrograde continuous flow			
Target scope (l/min)	Venc longitudinal (cm/s)	Venc non-longitudinal (cm/s)	Acquisition time (min:s)	Target scope (mmHg)	Venc longitudinal (cm/s)	Venc non-longitudinal (cm/s)	Acquisition time (min:s)
12	130	65	13:49	60	130	52	13:38
17	195	98	13:49	90	180	72	13:38
20	260	130	13:49	110	230	92	13:38
22	260	130	13:27				

### 2.3. Pulsatile flow setup

**FIGURE 3:**
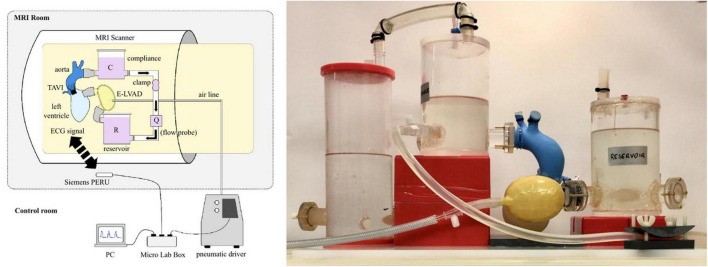
Schematic representation of the pulsatile flow setup **(left)** and photograph of the mock loop **(right)**.

For the pulsatile circuit with realistic flow and pressure patterns, the continuous flow circuit had to be substantially adapted. The pump was replaced with a pneumatic driving system (Thoratec^®^ dual drive console, Thoratec Laboratories Corporation, Berkley, CA, USA) outside of the MRI room and a pulsatile ventricle [ellipsoid heart, (15)] was placed directly in the MRI gantry (see Figure 3). Both devices were connected through the service port of the MRI room *via* an airline (polyester reinforced PVC tubing: length 10 m, inner diameter 6 mm).

The pulsatile circuit consisted of the same cardiac-aortic model including the TAVI, the pneumatic ventricle and the mock circuit, which was kept small to fit into the limited space of the MR scanner. Arterial afterload was modeled with a 2-element Windkessel (16) consisting of a metal-free 3D-printed tubular clamp and a closed acrylic reservoir (divided into two reservoirs to save space). An open reservoir was used to mimic atrial preload, and a 23 mm pyrolytic carbon bileaflet valve was used to replace the mitral valve.

The synchronization of the circuit with the MR scanner was done via a prototyping unit (MicroLabBox, dSPACE GmbH, Paderborn, Germany). The pressure signal was processed via the real-time interface and an ECG signal was sent wirelessly to the MR scanner (Siemens Physiologic ECG and Respiratory Unit, Siemens Healthineers AG, Munich, Germany), and then used to trigger the MR recordings (PMU).

In contrast to the continuous flow setup, the flow rate and pressure could not be measured directly during the MR measurement. Therefore, hemodynamics were adjusted in the control room (see Table 2) before final transport of the circuit into the MR scanner without the measurement equipment. Leaks or any circuit malfunctioning could be visually identified during the MRI acquisition via the fluid level in the mock loop visible at the patient camera of the MR scanner and changes at the pneumatic unit, but these did not occur. Hemodynamics were also verified in the control room once imaging was completed.

**TABLE 2:** Hemodynamic setpoints for the pulsatile circuit and adjustment of the pneumatic actuator.

	Parameter	Value
Mock loop	Cardiac output	5 l/min
	Aortic pressure (systolic/diastolic)	120/80 mmHg
Pneumatic drive	Systolic pressure	200 mmHg
	Diastolic pressure	−55 mmHg
	Systolic duration	30%
	Heart Rate	60 bpm

### 2.4. Magnetic resonance imaging compatible materials

One of the challenges of building setups for analysis using 4D MRI flow is the limited choice of MRI compatible materials. Therefore, plastics such as polyvinyl chloride, polylactic acid, and polymethyl methacrylate were used for the components used inside the scanner, additionally to silicone tubes, pyrolytic carbon valves, and non-ferromagnetic metallic materials. The materials used must not be pulled into the magnetic field of the scanner and their electromagnetic properties must not cause imaging artifacts especially in the areas of interest. In fact, non-ferromagnetic metallic materials such as brass, nickel and titanium, cause signal voids in their vicinity on MRI images.

This is also the case of the Portico™ valve stent, which is made of nickel and titanium alloy; indeed, according to technical specifications, it is compatible with a static magnetic field of up to 3 Tesla (17). However, this doesn’t mean that it doesn’t cause artifact; as a matter of facts, it attenuates the MRI signal within the stent so preventing a reliable MR signal acquisition. This is particularly challenging in the case of Portico™ valve, as it is equipped with a stent among the longest used for this application (49 mm), thus representing the worst case with regard to the presence of artifacts.

In this circuit numerous components, which do not have standardized connections were used. Therefore, several connectors, brackets and fasteners were created using 3D printing. For this purpose, both stereolithography printed (Clear Resin V4 of Formlabs 2, Formlabs Inc., Somerville, MA, USA) and fused deposition modeled parts (standard PLA material of Anycubic i3 Mega, Anycubic, Frankfurt, Germany) were used. In addition, a 3D printed support frame made of PLA was designed and created to achieve a phantom orientation similar to that of the left ventricle-aorta system observed on a patient lying supine on the MRI bed. To obtain a sufficient load for the MR coils, water bottles were positioned next to the circuit to achieve a realistic imaging scenario.

### 2.5. MRI acquisition and data evaluation

For the application of the 4D flow technique, magnitude and phase images were acquired using a Magnetom Prisma 3T scanner (Siemens Healthcare, Erlangen, Germany). A flexible receiver coil (Flex 4 Small, Siemens Healthcare, Erlangen, Germany) was placed on top of the flow phantom. The slices for the MRI acquisitions were positioned perpendicular to the ascending aortic tract in all measurements made. Since the different hemodynamic targets required different recording parameters and volumes of interest, different parameter sets were used, which are summarized in Table 3. A velocity encoded (VENC) 3D BEAT sequence was used for flow measurements. The VENC parameters were set based on the measured velocity so that there were no phase wraps, not only on expected jet velocities. To avoid velocity aliasing, long-axis scout sequences were used to improve and optimally adjust the original estimated values [10% higher than the estimated maximum speed parallel to the flow direction (18); see Table 4]. Pulsatile acquisitions were split into the systolic and diastolic phase to investigate the forward as well as the regurgitant flow.

**TABLE 3:** MR acquisition setting used for continuous antegrade and retrograde flow as well as for pulsatile systolic and diastolic flow.

MRI Parameter	Continuous antegrade flow	Continuous retrograde flow	Pulsatile flow for systolic phase	Pulsatile flow for diastolic phase
Spatial resolution [mm]	1.0 × 1.0 × 1.0	1.0 × 1.0 × 1.0	1.6 × 1.6 × 1.6	1.6 × 1.6 × 1.6
Rows	112	112	64	64
Columns	96	96	74	74
Slices	160	432	1600	440
Field of View [mm^3^]	116 × 99 × 160	116 × 99 × 432	115 × 100 × 1600	115 × 100 × 440
Echo time [ms]	3.45	3.13	2.79	2.87
Flip angle [°]	20	20	20	20
TR			45.2 ms	90.9 ms
Number of phases			20	9

**TABLE 4:** MR acquisition times and set velocity encoding rates used in the pulsatile flow acquisitions.

Pulsatile flow by systolic phase		Pulsatile flow per diastolic phase		
Venc *x* (cm/s)	550	Venc *x* (cm/s)	250	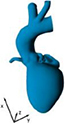
Venc *y* (cm/s)	300	Venc *y* (cm/s)	200	
Venc *z* (cm/s)	200	Venc *z* (cm/s)	150	
Acquisition time (min:s)	53:10	Acquisition time (min:s)	24:53	

The velocity encoded MR images were post processed using a previously developed (19) open source Python code,^1^ that allows the creation of flow maps, which are stored in a.vtk-file for each measurement. Continuous flow results were voxel-wise averaged over three consecutive measurements to improve the signal-to-noise ratio. Visualization of data was finally done with ParaView (Kitware Inc., Clifton Park, NY, USA), that additionally allows identification and isolation of regions of interests and calculation of flow rates.

### 2.6. Systolic and diastolic data evaluation

**FIGURE 4:**
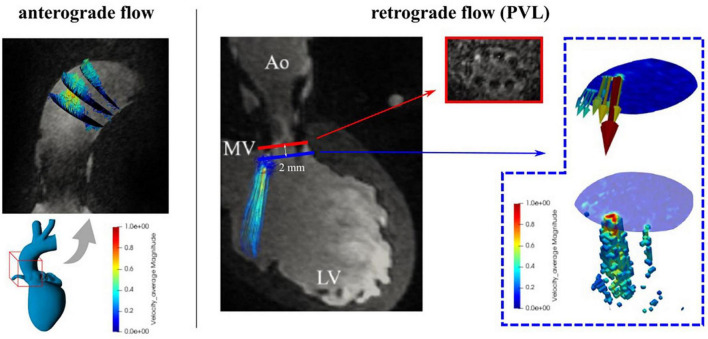
MR cross sections in the ascending aorta used for evaluation of antegrade **(left)** and retrograde **(right)** flow and paravalvular leaks (PVL) evaluation.

The systolic flow evaluation was performed using three equally spaced cross sections downstream of the valve inclined along the central axis of the aortic arch (see Figure 4). Aortic flow rates were calculated from both continuous and pulsatile measurements from the velocity vectors at the three cross sections and the three resulting values were averaged. Three-dimensional flow visualization using streamlines was limited to areas downstream of the valve prosthesis because of image artifacts within the valve stent. For this purpose, the corresponding areas were manually isolated in ParaView.

For analysis of diastolic flow, simulated by continuous retrograde flow and pulsatile flow, a cross section 2 mm downstream of the end of the stent in direction toward the ventricle was chosen, as close as possible to the aortic valve (see Figure 4). To identify the paravalvular flow rates, only velocity vectors between the 90th percentile (by visual identification) and the maximum velocity were used to calculate retrograde flow by multiplying it with the corresponding area. The three-dimensional representation of the PVL jet was done in a similar way: voxels that were part of the regurgitant jet were identified on the basis of a velocity boundary and the maximum velocity (98th percentile of velocity to avoid overestimation). For continuous flow settings the entire volume was averaged in three consecutive acquisitions to improve the signal-to-noise ratio. In addition, the kinetic energy content of the PVLs jet was calculated based on the equation $E_{k}=\sum_{i=1}^{N\,v\,o\,x\,e\,l}\frac{1}{2}\,\rho_{i}\,V_{i}\,v_{i}^{2}$, (ρ: density of the fluid, V*i*: volume of each voxel, *v*: is the velocity) which is a measure of the conversion of static to kinetic energy (18).

### 2.7. Ultrasound acquisitions

Verification of MR measurements was performed using routine clinical ultrasound (US) on the exact same model. An experienced cardiac surgeon performed color and CW Doppler measurements (S4-2 ultrasound probe, Affinity 50 ultrasound system, Philips, Eindhoven) and quantified paravalvular leakage and regurgitation immediately after MR measurements. The entire model was immersed in water to obtain better image quality and fewer artifacts. The ultrasound probe was positioned at the right coronary artery facing down toward the non-coronary sinus.

## 3. Results

Through the intensive use of 3D printing, an MR and US compatible realistic aortic model for the application of a TAVI valve was created, and both continuous and pulsatile flows could be studied and recorded using 4D MRI and echocardiography. The model represented only a portion of the arterial and aortic parts and therefore a normalized compliance was calculated for 100 ml of the model used. With a compliance of 0.28 ml/mmHg/100 ml, this was comparable to the values found in the literature (0.36 ml/mmHg/100 ml; 2.5 ml/mmHg for the entire 700 ml arterial system) (20, 21).

### 3.1. Continuous antegrade and retrograde flows

**FIGURE 5:**
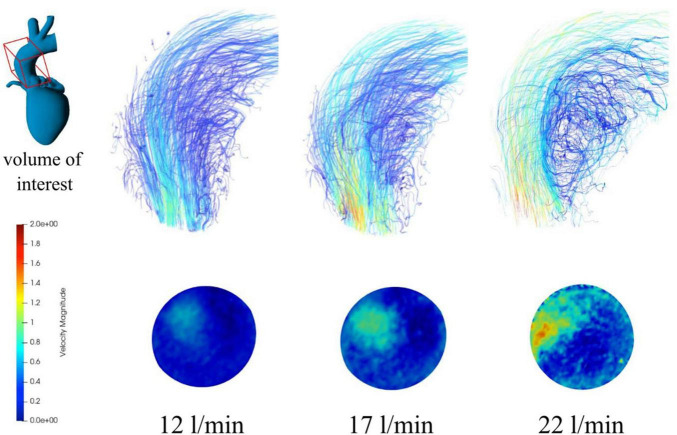
Streamlines **(top)** and contour maps **(bottom)** for continuous antegrade flow rates of 12, 17, and 22 l/min.

Continuous antegrade flow rates averaged over three sections showed a maximum deviation of 7% from the flowmeter measurements (see Table 5, for detailed data see Supplementary Table 1). Figure 5 shows the streamlines for flow rates from 12 to 22 l/min and the formation of a 3-dimensional vortex structure at the inner curvature of the aorta.

**TABLE 5:** Average continuous antegrade flow rates from the flowmeter and 4D flow.

Target flow rate [l/min]	12.0	17.0	20.0	22.0
Flowmeter flow rate [l/min]	11.5	17.1	20.7	22.0
4D MRI flow rate [l/min]	12.2	18.2	19.9	20.4
Error %	+6%	+7%	−4%	−7%

**FIGURE 6:**
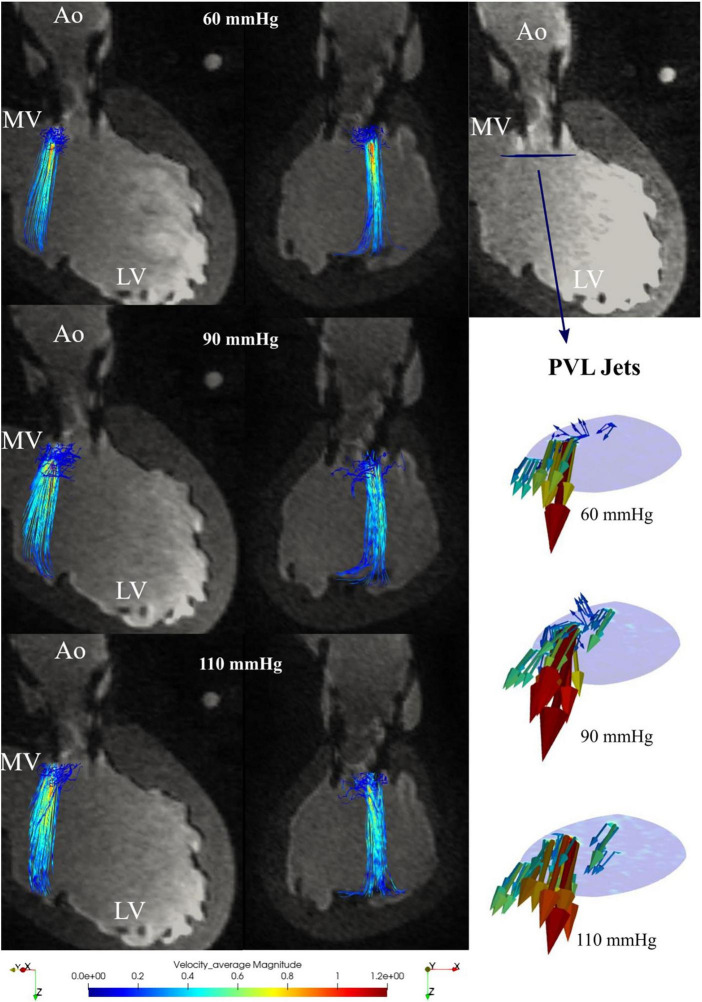
Retrograde paravalvular leaks (PVL) streamlines for 60, 90, and 110 mmHg transvalvular pressure in the 3-chamber view **(left)** and the xz-plane **(center)**; paravalvular jets in the plane directly below the transcatheter aortic valve implantation (TAVI) **(right)**.

Continuous retrograde flows were generated by applying transvalvular retrograde pressures, and by filtering and isolating PVLs based on velocity values both streamlines and individual PVLs could be identified and displayed locally (see Figure 6). In all cases one main jet was identified in the commissural position between the non-coronary cusp and the left coronary cusp. A second jet of smaller magnitude was also detected with all three applied pressures. The MR estimated flows (based on the 80th and 90th percentile) tended to slightly overestimate the flowmeter measurements (see Table 6).

**TABLE 6:** Continuous regurgitation flow rates measured with the flowmeter and 4D magnetic resonance imaging (MRI) quantification using different percentile boundaries.

Transvalvular pressure [mmHg]	60	90	110
Flowmeter flow rate [l/min]	−0.41	−0.56	−0.66
4D MRI flow rate (80 percentile) [l/min] (error from flowmeter [%])	−0.52 (+27%)	−0.80 (+43%)	−0.86 (30%)
Calculated MRI flow rate (90 percentile) [l/min] (error from flowmeter [%])	−0.46 (+12%)	−0.68 (+21%)	−0.59 (−11%)
V_max_ [m/s]	0.23	0.62	0.71
Kinetic energy [mJ]	0.030	0.040	0.039

### 3.2. Pulsatile flow conditions

The average pulsatile MR flow measurements showed a deviation of 18% (5.9 l/min, for more details see Supplementary Table 2) from the flowmeter measurement (5.0 l/min). Direct comparison with flowmeter measurements between systolic and diastolic flow was not possible because of geometric limitations, since placement of a flow probe at the position of the ascending aorta would have deformed the model and resulted in inaccurate flow measurements (22).

**FIGURE 7:**
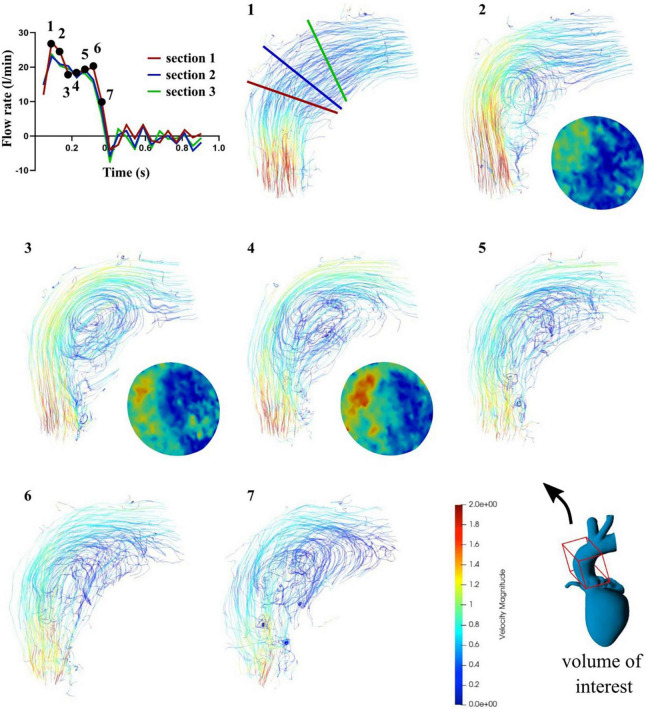
Flow rates (top left) and streamlines (1–7) in the case of pulsatile flow for seven systolic time frames and contour maps in section 2 for mid systole (2–4).

**FIGURE 8:**
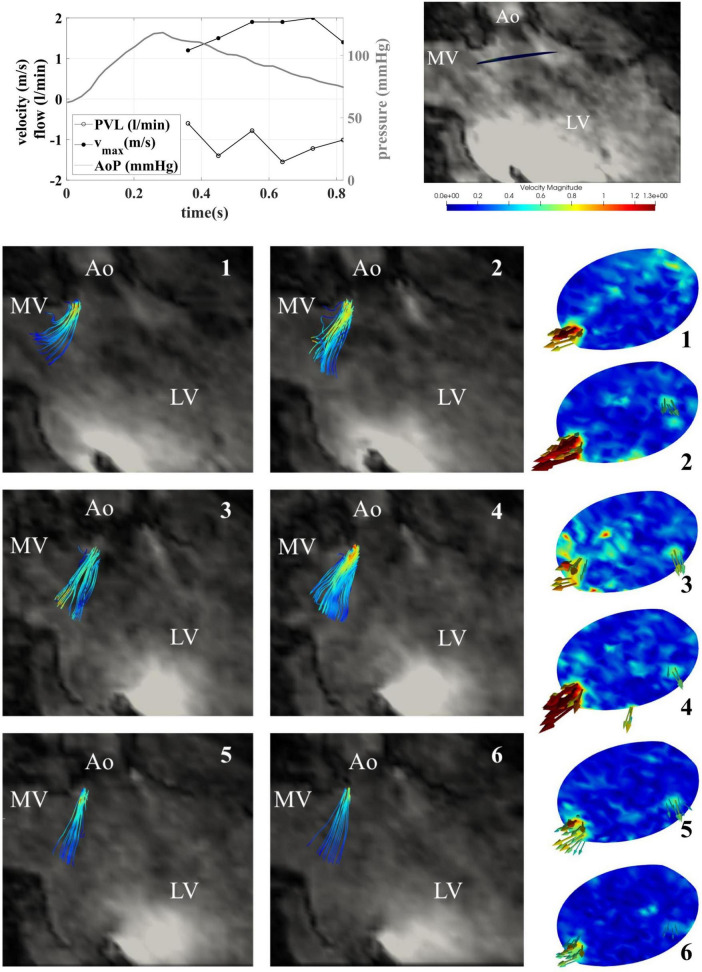
Retrograde pulsatile paravalvular leaks (PVL) streamlines shown for 6 diastolic timepoints in the 3-chamber view **(left)** and the xz-plane **(center)** and paravalvular jets in the plane directly below the transcatheter aortic valve implantation (TAVI) **(right)**.

**FIGURE 9:**
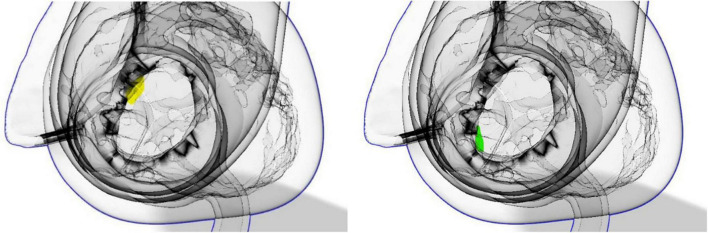
Location of PVLs in the case of continuous retrograde flow **(left)** and in the case of pulsatile flow **(right)** highlighted in yellow.

Flow patterns, calculated flow rates, peak velocities and PVL visualizations were possible for seven time points of the systolic phase (see Figure 7) and six time points in the diastolic phase (see Figure 8). Within time frame 3 some artifacts were captured and regurgitant flow rates were underestimated at this time point, whereas the peak velocities reached a maximum. Nevertheless, the regurgitant volume, calculated as an integral of the retrograde flow curve, was calculated to be about 10 ml per beat and classified as “mild” on the 5-degree scale for PVLs (23). Additionally, the main PVL jet was not completely in the same position as in the retrograde continuous flow study, but at the position of the non-coronary cusp, as shown in Figure 9.

### 3.3. Ultrasound acquisition results

**FIGURE 10:**
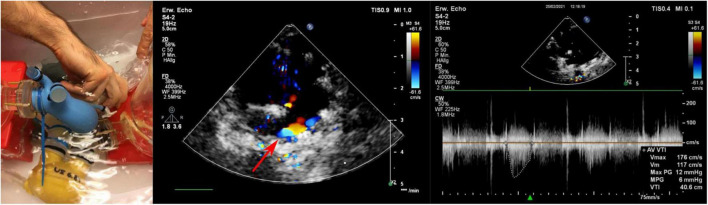
Echocardiographic acquisition **(left)** and determination of PVLs by Doppler **(middle)** and continuous wave **(right)** measurements.

Using color Doppler ultrasound measurements in the pulsatile circulation, a single PVL jet was identified at 6 o’clock at the non-coronary cusp (see arrow in Figure 10) which was consistent with the MR measurements. For the maximum velocities, similar values were found in ultrasound (1.7 m/s) and MR (2.0 m/s) and the PVLs were classified as “mild” (23) by an experienced cardiac surgeon identical to what was seen in the MRI.

## 4. Discussion

Using 3D printing techniques, a realistic phantom of the left ventricle, aortic root, and ascending aorta could be created, allowing qualitative and quantitative measurements by 4D MRI not only in antegrade flow but also in retrograde flow when using a TAVI valve. While other TAVI valves have been used for similar purposes in MRI (24, 25) the Portico valve presents a particular challenge in terms of image artifacts because of its large stent. However, the resulting imaging artifacts remained limited to the area where the metallic stent structure was located.

During antegrade flow, the formed vortex at the inner curvature of the aortic root, which was even more pronounced during peak systole with pulsatile flow and was accompanied by maximum flow velocity at the outer curvature of the aorta, as has also been described previously in the literature (26, 27). This marked severe eccentricity of flow with peak velocities at the outer curvature of the ascending aorta is a non-physiological phenomenon and has been also noted *in vivo* after transcatheter valve implantation in up to 43–65% of patients (26, 27).

Evaluation of the paravalvular leaks by 4D MRI allowed clear visualization of the number, the direction, and the location of PVLs below the valve, with no central regurgitation jet. Although the position of the PVLs was slightly different in continuous and pulsatile flow, the regurgitation jets were consistent in the MRI and US examinations. The difference in position of the regurgitant jets between the two cases is most likely due a slightly different valve positions in the two different experimental setups, as they could not be performed on the same day because or MRI availability and the valve had to be repositioned. Markings on the outside of the model therefore seem too imprecise for exactly the same positioning to be possible. In addition to the increased cardiac work-load as a consequence of PVLs, the position of the PVL jets is an important issue, as it also influences formation of ventricular vortices and thereby might further adversely effect the ventricular ejection (11).

The maximum measured velocities of the PVLs by MRI and US were almost comparable. The maximum of 2.0 m/s measured by MR was only 18% higher than the US measurements of 1.7 m/s. Both imaging techniques suffer of limitations; MR doesn’t allow the jet measurement directly in the valve due to artifacts (28); the US measurement is strongly position dependent and might not be very accurate for small jets. However, both methods clearly show that larger pressure gradients are associated with higher PVL flows. This relationship is in agreement with previous studies between mean aortic pressure and observed amount of PVLs (3, 29). Even considering the limited local resolution [MR voxel size 1.6 mm, US slice thickness few millimeters (30)] of the two methods, the results are comparable and satisfactory. Due to these limitations, accurate calculations of PVL areas and flow is not achievable.

The initial assessment of retrograde flow from the PVLs was done using the 90th percentile of the velocity as the threshold value for filtering. However, for 60 and 90 mmHg, the flow rate was overestimated compared with flowmeter measurements and for the application of 110 mmHg, on the other hand it was underestimated by 11%. Indeed, it seems logical that as the velocities of the PVLs increases, the retrograde flow also rises. Therefore, the threshold was lowered to the 80th percentile of velocities (Table 6), which resulted in a general overestimation of PVL flow by an average of 33%. The reason for this overestimation is that the assessment had to be made downstream of the jet origin because of imaging artifacts directly at the valve plane. The jet entrained some of the fluid contained in the ventricle similar to what happens in a jet pump, where a deliberately generated jet is used to create flow by entraining surrounding liquid. This also happened in the ventricle with the PVL jets and resulted in an overestimation of the PVL flow rates. Using higher thresholds did not allow to capture this jet dynamics at high pressures. Therefore, this did not directly correspond to the actual flow rate passing through the holes of the paravalvular leaks and should be considered in a clinical evaluation. Thresholding might further depends on the PVL geometries and cannot be generalized. Nonetheless, all these effect should be systematically investigated in the future.

The circuit was designed so that PVL should occur. For this purpose a considerable ellipticity was chosen, which is known to weaken the performance of self-expanding transcatheter aortic valves (31). The eccentricity index (EI = 1-D_min_/D_max_), which was calculated from the minimum and maximum ring diameters (D_min_, D_max_), can be used to determine this deviation from a circular shape and a value of 0.26 was calculated for the given geometry. Values greater than 0.25 are associated with a significant increase in paravalvular regurgitation and fits to the findings within this study (32).

Knowledge of the interaction of devices with cardiac structures is important to understand the mechanisms that lead to device malfunction, damage, or damage to cardiac structures and are absolutely essential to the success of therapy. This work provided a first step in the study of TAVI valves and flow-associated complications and may be of particular interest to patients with complications in the near future. With further refinements to the measurement protocols, it is certainly possible to reduce recording times to a level that is also clinically feasible.

### 4.1. Limitations

The chosen model cannot fully represent the complex physiological interaction of myocardial contraction, aortic compliance and a TAVI valve. However, by using elastic silicone, a certain degree of elasticity was incorporated, which may have contributed to vortex formation due to the cross-sectional change in the aortic root by the systolic distension. In addition, the ventricular model itself was non-contractile, but pulsatile flow moved through the model. However, this does not directly affect PVL formation and a contractile ventricle could be realized in the future by adjusting the mock loop. The positioning of the TAVI valve in the aortic root could be improved. Although the stent structures were marked, the position changed during conversion to the pulsatile configuration.

The pulsatile 4D MRI flow estimation could not be validated by flowmeter measurements as a flow probe on the elastic aorta would have restricted the systolic distension and would have resulted in inaccurate flow readings. Another inherent restraint of MRI flow measurements is caused by its limited resolution. Thereby it is currently almost impossible to resolve turbulent flow structures in the sub millimeter region and temporal changes (33).

Magnetic resonance imaging is not currently an integral part of the clinical workflow in TAVI patients and is typically more costly in terms of money and time, although it offers some potential to improve efficiency in terms of time and quality. However, MRI provides much more information on flow patterns (27, 34) and may be worth integrating into future follow-up (35) to identify flow related complications.

## 5. Conclusion

The use of 3D printing methods allowed the application of 4D-MRI flow technique and enabled promising results in the detection of PVLs and proved to give better insights into flow behavior compared to ultrasound images, as it also provided the possibility to visualize paravalvular jets in their three-dimensionality in a qualitatively accurate way with a tendency of overestimating regurgitant flows. Accurate quantification of PVLs was not always possible because direct measurements in the valve were not possible due to image artifacts. Clinical application of 4D-MRI in TAVI patients is possible but is still limited by financial and time restrictions.

## Data availability statement

The original contributions presented in this study are included in the article/Supplementary material, further inquiries can be directed to the corresponding author.

## Author contributions

FM, AR, MM, and PA contributed to conception and design of the study. ES, SP, TK, MM, MK, and PA developed and performed experiments. ES, SP, TK, MM, and SS analyzed the data. All authors contributed to manuscript revision, read, and approved the submitted version.

## Footnotes

1
https://github.com/saitta-s/flow4D
