# Supplementary material for: Quantification of paravalvular leaks associated with TAVI implants using 4D MRI in an aortic root phantom made possible by the use of 3D printing
Source: Front Cardiovasc Med. 2023 Jan 19;10:1083300. doi: 10.3389/fcvm.2023.1083300 (PMC9894656; doi:10.3389/fcvm.2023.1083300)
Supplement: Supplementary file 1 [file Table_1.docx]

Supplementary Material

# Supplemental Tables

Supplementary Table 1: Target flow rates, measured flow rates by the flowmeter and 4D MRI flow rates in three cross sections

|  | **Target**  **Flow** | **Measured Flow (Flowmeter)** | **Average Flow** | **4D Flow Section1** | **4D Flow Section2** | **4D Flow Section3** | **Average 4D Flow** | **Error** |
| --- | --- | --- | --- | --- | --- | --- | --- | --- |
|  | **[l/min]** | **[l/min]** | **[l/min]** | **[l/min]** | **[l/min]** | **[l/min]** | **[l/min]** |  |
| Meas. 1 | 12.0 | 11.46 | 11.5 | 12.40 | 12.06 | 12.08 | 12.2 | +6% |
| Meas. 2 |  | 11.50 |  |  |  |  |  |  |
| Meas. 3 |  | 11.48 |  |  |  |  |  |  |
| Meas. 1 | 17.0 | 17.04 | 17.1 | 18.80 | 18.10 | 17.80 | 18.2 | +7% |
| Meas. 2 |  | 17.02 |  |  |  |  |  |  |
| Meas. 3 |  | 17.20 |  |  |  |  |  |  |
| Meas. 1 | 22.0 | 22.00 | 22.0 | 20.51 | 20.73 | 20.02 | 20.4 | -7% |
| Meas. 2 |  | 21.95 |  |  |  |  |  |  |
| Meas. 3 |  | 22.00 |  |  |  |  |  |  |

Supplementary Table 2: Flow measurements of the pulsatile setup for systole analysis (group C), obtained in all three sections from the post-process with 4D flow.

| **Time [s]** | **4D Flow QSection1 [l/min]** | **4D Flow QSection2 [l/min]** | **4D Flow QSection3 [l/min]** |
| --- | --- | --- | --- |
| 0.0452 | 12.0 | 14.8 | 15.0 |
| 0.0904 | 27.3 | 23.1 | 23.7 |
| 0.1356 | 24.4 | 20.8 | 20.4 |
| 0.1808 | 18.1 | 20.3 | 19.5 |
| 0.2260 | 18.3 | 17.0 | 18.2 |
| 0.2712 | 19.2 | 19.2 | 18.1 |
| 0.3164 | 19.9 | 16.3 | 15.5 |
| 0.3616 | 10.2 | 7.9 | 6.1 |
| 0.4068 | -4.1 | -5.8 | -7.5 |
| 0.4520 | -2.6 | 0.3 | 2.0 |
| 0.4972 | 3.3 | 1.5 | -0.3 |
| 0.5424 | -0.7 | -3.2 | -3.8 |
| 0.5876 | 3.3 | 2.9 | 3.0 |
| 0.6328 | -1.4 | -2.5 | -3.4 |
| 0.6780 | -1.9 | -0.7 | 1.4 |
| 0.7232 | 1.5 | 0.1 | -0.9 |
| 0.7684 | -1.9 | -2.2 | -0.7 |
| 0.8136 | 1.8 | 0.8 | 0.2 |
| 0.8588 | -0.5 | -4.3 | -2.8 |
| 0.9040 | 0.7 | -1.9 | -0.5 |
| 0.9492 | 0.0 | 0.0 | 0.0 |
| 0.9944 | 0.0 | 0.0 | 0.0 |
| **Average flow rate [l/min]** | **6.67** | **5.66** | **5.61** |
